# Motivational interviewing - from theory to practice.

**DOI:** 10.1192/j.eurpsy.2023.191

**Published:** 2023-07-19

**Authors:** A. R. Moura

**Affiliations:** he Max Planck UCL Centre for Computational Psychiatry and Ageing Research, London, United Kingdom

## Abstract

**Abstract:**

In this fourth session of our motivational interviewing workshop, we will go from theory to practice through simple and direct examples from clinical practice. The participants will be able to watch previously filmed clinical cases and participate in role plays, where they can practice using different motivational interviewing techniques that they had learned before.

**Disclosure of Interest:**

None Declared

